# School-Life Origin of Nervous Diseases

**Published:** 1877-04

**Authors:** P. H. Hayes


### THE SCHOOL-LIFE ORIGIN OF NERVOUS DISEASES.

P. H. HAYES, M. D.

I was this morning told a shocking story by one of the young lady teachers in our public schools. She said "a little girl in one of my classes had the habit of clinging to me and leaning upon my lap whenever she could get an opportunity. Observing her more closely I saw that it seemed impossible for her to stand still or to hold a book still, and often her hands and arms would jerk and her book would fall in spite of her efforts to hold it." This teacher adds, "I spoke to the little girl about the twitching, and she said that last Sunday her mother whipped her because she let her plate fall as she was passing it for food." At the close of school this teacher went home with her little protege and told the mother her child needed a physician and not the rod.

Dr. Allen McLane Hamilton declares as a result of his investigations in the public schools of New York, that twenty-two out of every one hundred of the children twitch their hands or their faces on one side of the body, and that twenty-five out of every one hundred are subject to headache. Again it was found upon inquiry that only two per cent. of those who suffered from twitching, and only ten per cent. of those who suffered from headache were found among the higher grades of the school. Now the intelligent inference here is not that these forms of nervous disease have largely been recovered from as the children advanced from the primary to the grammar grades, but that the children have fallen out of the ranks entirely and been compelled to leave school. And again the conclusion is almost indisputable, that these diseases in the grammar grades are of very recent origin and that these unfortunate pupils must soon in their turn, fall out of the ranks and leave the school.

I have mentioned only two forms of the nervous diseases of children, but I hardly need say to those who have closely observed the school-life of children that there are many others that begin with their school years. Most common of all is indigestion leading to imperfect nutrition and of course to imperfect growth and development. Indeed, all the nervous diseases I have met with among school-going children were only the symptoms of exhaustion, and that exhaustion was brought on by excessive study, excessive anxiety to keep up with classes and to stand high, and if possible highest, in coming competitive examinations. I must not omit to state that the often bad air of the school-room must contribute largely to the exhaustion I have mentioned. But all of these causes would fail to bring on ill-health in vigorous girls and boys if they were not allowed, as they often are, nay, put forward by their parents, to take music, or drawing lessons, or embroidery, out of school hours, and thus overtax the brain. Moreover, such a load of brain-work often compels late hours and insufficient sleep, and what wonder now that our boys have headaches and our girls St. Vitus dance, hysteria and indigestion by the time they are well in their teens.

Moreover, the magnificient libraries of which our public schools are so proud, are often a great damage to pupils in the schools. I know of boys and girls scarcely in their teens who go regularly to these libraries and select exclusively such books as arouse and excite their imagination and the emotional nature, and just as soon as books of study can be laid aside, even for a moment, they take up those books and pore over them at every opportunity and often till long after they should be in bed. How often have I been told by mothers in consultation, "all Sarah wants is to get a book and get off by herself." These books fill the minds of children with reveries which they greatly enjoy, but which exhaust the brain and render them irritable. The truth is these reveries, this exercise of the imagination, has become almost necessary to their happiness and they do not like to be interrupted.

*Exercise*, we say, develops and strengthens every part of the body. But this is true only in a limited sense as to the nervous system. When this is exercised so constantly and without the balancing and bracing effects of out-door sports and the full restorative power of sleep, its activity and its sensitivity are greatly increased, but it neither grows nor is it strengthened. There cannot be growth and lasting vigor of brain without abundant nutrition, and there cannot be abundant nutrition without abundant digestion, and there cannot be abundant blood-making without more out-door life and the free and joyous exercises which are both instinct and necessity for all child-nature.

An intelligent mother right here in our midst, went a short time since to visit her son in college. This son was strong and of a fair and ruddy complexion, and felt a wholesome pride in keeping up a fine physique and thus having body and brain go hand in hand in their development. His mother felt a little anxious as to his scholarship and ventured to ask him if he was not giving most too much time to his walking and other exercises. Now, said he, mother don't you want to see the young man who has taken first prizes here for the last three years? Yes, she said she did. Well, said he, do you see that pale, thin young man, with a form like a tuning fork, just coming into the hall? That is he—mark him well. That young man wears two flannel shirts and three pairs of drawers to keep himself comfortable in the winter, and he very prudently keeps close in the college when there is any inclement weather, and especially if there is a high wind outside! That young man, mother, with his last lessons in Horace is taking his first lessons in dyspepsia and the "*Hypo.*" There is certainly everywhere a false estimate of the value of book-knowledge. The scholar prides himself on the number of books he has crammed into his brain, and the young lady that she has not only kept up with her classes but she has learned besides, out of school-hours, music, drawing, and embroidery. Superadded to our common human nature, has every young miss in school, her woman nature, which through all these years of school-life is progressing through an evolution around which gathers the charms of life and the glory of pefect womanhood. Yet how often do I now find among my consultations, young women in whom this evolution is retarded and rendered painful and incomplete by high-pressure systems of education. Does not nature and history everywhere teach us the broad lesson that *strength* is necessary to the crownings and rejoicings of life, and that to be weak is to be miserable? I remember well two young ladies who came under my care at the Sanitarium at C—— S——, some years ago. They had just graduated at a metropolitan seminary, and so important did this event seem to them that all subsequent and even long previous events were dated from this epoch—so long before or so long after they graduated at M——. They appeared to have large knowledge of books; they were well trained in music and almost to affectation in the proprieties. They were girls of real spirit, and felt as I think, there was a grand blunder somewhere, that fresh with academic honors, they should have made their *entree* into life by going into a sanitarium for chronic invalids. Their mother who accompanied these young ladies, was strong and healthy and able to lead about and be servant to her tender daughters. These were dyspeptic and bloodless, their fate (?) *invalidism. Cui bono?* Here were learning and accomplishments and in the getting neglect of exercise and loss of health, by which alone they could be used and enjoyed. They did not seem to have any natural vivacity, nature was schooled out of them. They went about exercise as though it were duty, something ecclesiastical, judicial or expiatory. They had no natural sense of fatigue, therefore no real sound and refreshing sleep. They had, of course, no genuine sense of hunger, the true seasoning for our food. They came down in the morning with the *bouche pateuse* or clammy mouth, and they would look wistfully around the table for some sharp sauce to quicken appetite; "*can't* eat—everything tastes so horribly." Is it within the art of doctors to restore, in a few months, as this mother expected me to do, the waste of the powers and actions of life which had been going on for thirteen years? I trow not.

